# Maintenance of Effects and Correlates of Changes Following Mindfulness for Interdisciplinary Health Care Professional Students

**DOI:** 10.1089/imr.2021.0012

**Published:** 2022-09-07

**Authors:** Sarah Ellen Braun, Samantha N. Mladen, Christina M. Luberto, Patricia Anne Kinser

**Affiliations:** ^1^ Division of Neuro-Oncology, Department of Neurology, School of Medicine, Virginia Commonwealth University, Richmond, VA, USA.; ^2^ Massey Cancer Center, Richmond, VA, USA.; ^3^ Department of Psychology, Virginia Commonwealth University, Richmond, VA, USA.; ^4^ Department of Psychiatry, Massachusetts General Hospital/Harvard Medical School, Boston, MA, USA.; ^5^ School of Nursing, Virginia Commonwealth University, Richmond, VA, USA.

**Keywords:** mindfulness, stress, burnout, health care professional students, activity impairment, RCI

## Abstract

**Objectives:**

To evaluate long-term outcomes after an 8-week mindfulness intervention, Mindfulness for Interdisciplinary Health Care Professionals (MIHP), and investigate relationships between outcomes overtime.

**Design/Methods:**

In this single-arm study, 35 participants received MIHP and completed measures of burnout, perceived stress, activity impairment, and dispositional mindfulness at baseline, post-MIHP, and a 3-month follow-up. Changes over time were evaluated using repeated-measures analysis of variance (ANOVA) and reliable change indices (RCIs). Then, correlations between dispositional mindfulness and distress/impairment outcomes were evaluated.

**Results:**

At follow-up, aspects of burnout and several mindfulness skills demonstrated maintained improvements. RCIs showed that a higher percentage of participants improved on all outcomes at each time period than declined—all outcomes showed little to no deterioration. However, most participants did not reliably change, and this was more pronounced at the follow-up. Changes in two mindfulness skills (acting with awareness and nonjudging of inner experience) were consistently negatively correlated with distress and impairment outcomes.

**Conclusions:**

Acquired mindfulness skills during MIHP were maintained at the follow-up. RCI analyses demonstrated that MIHP may protect against worsening stress and burnout during training. Two mindfulness skills, acting with awareness and nonjudging of inner experience, showed potential mechanistic effects on work-relevant outcomes. Booster sessions to encourage maintained mindfulness practices and skills should be investigated in future trials. This study was registered on clinicaltrials.gov (#NCT03403335) on January 11, 2018.

## Introduction

Stress and burnout in health care professional (HCP) students have been well documented.^1,2^ Stress refers to the perception that environmental demands exceed coping resources.^3^ Burnout is a specific type of work-related stress, and is characterized by emotional exhaustion, cynicism, and a decreased sense of personal accomplishment at work.^4^ Stress and burnout occur at much higher rates in HCPs than the overall population,^5^ and evidence suggests that it begins in training,^2^ with 54% of HCP students endorsing elevated levels of both.^6^ Stress and burnout are associated with decreased empathy,^7^ increased substance use,^8^ and during training, with more unprofessional behaviors and a higher likelihood of cheating.^9^

A large body of evidence documents the deleterious effects of chronic stress on brain-based measures of performance and cognition.^10^ Similarly, a growing body of research has documented an association between burnout and poorer work performance^11,12^; decreased cognitive function^13,14^; and, poorer patient outcomes.^15^ Overall, stress and burnout in HCPs have been estimated to be costly on the health care system, warranting attention to intervention and prevention strategies.^16^

Responding to calls to address the so-called “burnout epidemic,” research investigating mindfulness-based interventions has grown in the recent decade.^17,18^ There are many different conceptual definitions of mindfulness; here, following the widely used conceptualization of Baer et al., we define it as a multifaceted construct comprised of five facets, or skills: (1) observing—noticing and attending to stimuli in the internal and external environment; (2) describing—verbally labeling inner experiences; (3) acting with awareness—intentional awareness of oneself in the present moment, the opposite of automatic pilot; (4) nonjudging of inner experience—a nonjudgmental attitude toward thoughts and feelings; and, (5) nonreactivity to inner experience—the ability to let inner experience come and go without reaction or entaglement.^19^

Meta-analytic evidence demonstrates increases in these skills following mindfulness-based interventions relative to active control groups.^20,21^ This study utilized the five facets of mindfulness as the operationalization of mindfulness and as an important measure of acquired skills following intervention.

Although preliminary research supports the efficacy of mindfulness interventions to reduce stress and burnout in health care providers and students,^17^ more rigorous research designs have been called for to enhance the science. For example, studies are warranted, which evaluate longitudinal outcomes, with follow-up time points beyond the end of the intervention, and that investigate mechanisms by which mindfulness intervention may improve these outcomes.^17^

Mindfulness for Interdisciplinary Health Care Professionals (MIHP) is a recently developed program that applies the principles and practices of mindfulness to the specific stressors faced by HCPs and students.^22^ A recent partially randomized controlled trial (RCT) of MIHP found decreased burnout, stress, and daily activity impairment alongside increased dispositional mindfulness among the MIHP group relative to the control group in a sample of interdisciplinary HCP students.^23^

Another earlier investigation of MIHP using a single-arm design found maintained effects of MIHP on burnout, stress, and dispositional mindfulness in a small sample of HCPs 1 year following the intervention.^24^ However, these, and the majority of previous studies on mindfulness for HCPs have relied on analyses at the group level to quantify changes in burnout, stress, and other relevant outcomes. Very little research has been conducted at the individual level,^25,26^ calculating the percentage of participants who improve, remain stable, or deteriorate overtime.

Investigation of clinically significant change in relevant outcomes is an important achievement to establish before conducting larger scale RCTs.^27^ Analyses at the individual level could provide more nuanced information regarding the effects of MIHP, without which important subtleties regarding subgroups of participants with different responses to the intervention may be missed.^28^

Moreover, very little research has explored associations between changes in mindfulness skills with burnout and stress among HCP students in mindfulness interventions.^29^ Understanding these potential associations is needed to inform future mechanistic studies as well as provide insight on how specific qualities of mindfulness are associated with stress and burnout in HCPs. There is empirical and theoretical evidence to suggest that dispositional mindfulness may be a driver of the positive changes seen postintervention.^20,30,31^

The first aim of this study was to replicate findings of maintained postintervention effects^23^ at a 3-month follow-up among students in MIHP. Outcomes of interest were burnout, perceived stress, daily activity impairment, and mindfulness skills. It was hypothesized that each outcome, which showed improvement from pre-postintervention in a previous paper,^23^ would show maintained improvement at follow-up (e.g., maintained significant improvement from baseline to follow-up).

Then, to thoroughly assess possible intervention effects at the individual level, reliable change indices (RCIs) were calculated to characterize the sample in terms of responder status at postintervention and follow-up. The second aim of this study was to explore correlations between changes in mindfulness skills and each outcome over the same time range (e.g., baseline to post-MIHP or post-MIHP to follow-up). It was predicted that change on all facets of mindfulness (acting with awareness, nonjudging of inner experience, nonreactivity to inner experience, observing, and describing) would be negatively associated with change in burnout, stress, and daily activity impairment.

## Methods

### Procedure and design

This was a single-arm study resulting from a crossover design. After eligibility confirmation, participants were scheduled for a baseline assessment where informed consent was obtained, and they were randomized to either MIHP or the waitlist control. Preferential group allocation was offered to participants in cases when their class/clinical schedules did not allow them to attend the intervention to which they were randomized, resulting in a partially randomized design (a total of 14 treatment completers were randomized, and 21 preferentially allocated according to schedule). First, the mindfulness group received MIHP. Then, following a crossover design, the waitlist control participants received MIHP. Three months after the completion of MIHP, participants were asked to complete follow-up measures.

**FIG. 1.**
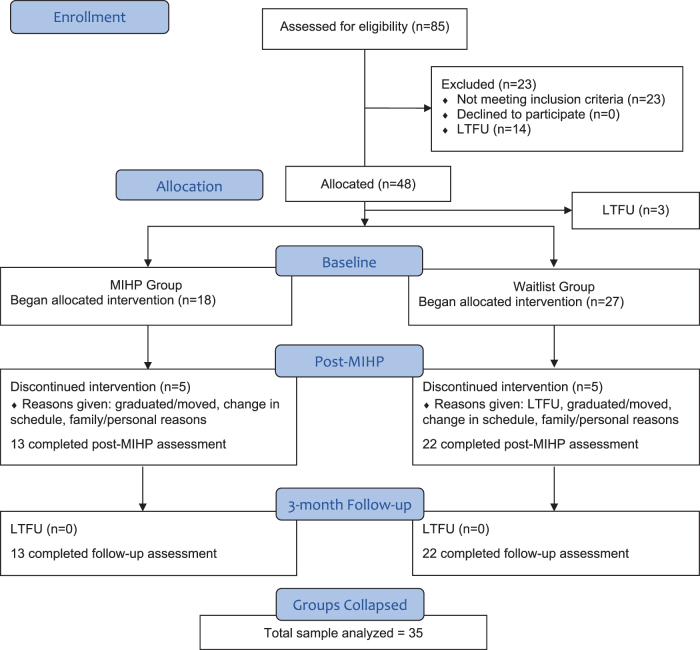
CONSORT flow diagram. LTFU, lost to follow-up; MIHP, Mindfulness for Interdisciplinary Health Care Professionals.

Participants were then collapsed into one group with three time points of interest: baseline, post-MIHP, and follow-up. Groups were collapsed so that the time intervals were equivalent and corresponded to the period during which each group completed MIHP; that is, 8 weeks elapsed between baseline and post-MIHP for both groups and 3 months elapsed from post-MIHP to follow-up for both groups (see Fig. 1 for participant flow). This study was approved by the Institutional Review Board at Virginia Commonwealth University (HM20012397), and was performed in accordance with the ethical standards as laid down in the 1964 Declaration of Helsinki and its later amendments.

### Participants

Participants were 35 HCP students who enrolled and completed MIHP.^23^ For in-depth information on the MIHP curriculum and study protocol, see Braun et al.^23^

#### Inclusion criteria

Participants were ∼18 years of age, enrolled as a student in one of the following disciplines: School of Nursing, School of Dentistry, School of Pharmacy, School of Medicine, Clinical or Counseling Psychology graduate programs, Social Work graduate program, Allied Health, or undergraduate students with an 80% certainty they would apply to graduate school in a health care profession.

#### Exclusion criteria

Individuals who self-reported engagement in a consistent mindfulness-based activity (such as yoga or meditation) more than once per month for the past 6 months were excluded.

### Measures

#### Burnout

The Maslach Burnout Inventory—Student Survey (MBI)^32^ was used to assess student burnout. It is a 16-item questionnaire of burnout symptoms falling on three subscales: exhaustion, cynicism, and professional efficacy (reverse scored). Respondents rate items on a seven-point scale from “not at all” to “everyday.” This is the gold standard of burnout measurement in HCP student samples and has been widely used, including several translation and validation studies.^33–40^ Scores on the cynicism subscale should be interpreted as follows: low 0–5; moderate 6–10; high ≥11. Scores on the exhaustion subscale should be interpreted as follows: low 0–10; moderate 11–15; high ≥16.

#### Perceived stress

The Perceived Stress Scale (PSS-14)^41,42^ is a 14-item survey with a 5-point scale ranging from “never” to “very often” assessing symptoms of stress over the last month.

#### Work/school productivity

The Work Productivity and Activity Impairment Questionnaire plus Classroom Impairment Questions (WPAI+CIQ) assessed hours missed of work and school, impairment at work and school, as well as daily activity impairment due to physical and mental health issues in the last 7 days.^43,44^ This questionnaire provided four scores of interest: (1) absenteeism or the sum of hours missed from work and school relative to hours scheduled; (2) presenteeism or average level of perceived impairment in work and school; (3) presenteeism in daily activities; and (4) work productivity loss, which is the aggregate of absenteeism and presenteeism in work and school.

#### Dispositional mindfulness

The Five Facet Mindfulness Questionnaire (FFMQ)^19^ is a 39-item survey with a 6-point scale ranging from “Never, or very rarely true” to “Very often or always true” assessing one's tendency to be mindful in daily living. The scale is comprised of five facets, or subscales: acting with awareness, nonjudging of inner experience, nonreactivity to inner experience, observing, and describing.

### Data analysis

Repeated-measures analyses of variance (ANOVAs) were used to test for differences across the three time points for all measures. *Post hoc* pairwise comparisons were adjusted using Bonferroni, and the significant level for this study was set at *p* < 0.05. RCIs were then calculated for each variable of interest (two scales of burnout, perceived stress, and five facets of mindfulness) from baseline to post-MIHP and from baseline to follow-up.

RCI was not calculated for work impairment, as it is a single item. These scores were calculated for each participant by dividing the change over time by the standard error of measurement, a value calculated from published standard deviations and Cronbach's alpha for each measure, respectively.^19,45,46^ Each participant was then categorized as having reliably increased, reliably decreased, or not changed on each variable of interest based on whether the RCI score fell below, within, or >1.96 standard errors of measurement.^47^

To analyze change score correlates, first, group-level change scores were calculated from baseline to post-MIHP, from post-MIHP to follow-up, and from baseline to follow-up. Change scores were calculated for each variable of interest (two scales of burnout, perceived stress, daily activity impairment, and five facets of mindfulness) by subtracting the earlier time point from the later time point score. Bivariate point-serial correlations were then calculated for each pair of outcome and mindfulness variables across the same time range (e.g., act aware and perceived stress from baseline to post-MIHP, act aware and work impairment from post-MIHP to follow-up, etc.). All analyses were conducted in SPSS Version 26.

## Results

Demographics for this sample are presented in Table 1. Results from repeated-measures analysis of variances pairwise comparisons revealed a significant improvement from baseline to post-MIHP for exhaustion, cynicism, perceived stress, activity impairment, and all five facets of mindfulness. Improvements are interpreted as decreases on measures of burnout, perceived stress, and activity impairment and as increases in mindfulness skills (see Table 2).

**Table 1.** Sample (*n* = 35) Demographic Information

Demographic variable	*n*	Percentage or M* (*SD)
Gender		
Female	33	94.3
Male	2	5.7
Race/ethnicity		
White/European American	20	57.1
Asian/Asian American/Pacific Islander	5	14.3
Black/African American	6	17.1
Latino/Hispanic	1	2.9
Other	2	5.7
Missing	1	2.9
Marital status		
Single	26	74.3
Living with partner/married	7	20.0
Separated/divorced/widowed	1	2.9
Missing	1	2.9
Age		27.4 (7.9)
Current level of education		
Undergraduate (prehealth or nursing)	12	34.3
Graduate	23	65.7
Discipline		
Nursing	12	34.3
Medicine	11	31.4
Allied health	3	8.6
Psychology	3	8.6
Dentistry	2	5.7
Pharmacy	2	5.7
Social work	2	5.7

SD, standard deviation.

**Table 2.** Repeated-Measures Analysis of Variance—Pairwise Comparisons

Measure	Baseline, M (SE)	Post-MIHP, M (SE)	Follow-up, M (SE)
Exhaustion (*n* = 35)	16.1 (1.2)	12.4^a^ (1.4)	12.8^a^ (1.4)
Cynicism (*n* = 35)	10.2 (1.2)	7.9^a^ (1.1)	10.5^b^ (1.2)
Perceived stress (*n* = 31)	26.6 (1.3)	20.8^a^ (1.3)	24.3^b^ (1.6)
Activity impairment (*n* = 32)	28.9 (5.5)	14.3^a^ (3.8)	21.1 (4.4)
Acting with awareness (*n* = 32)	23.9 (0.8)	26.9^a^ (0.8)	26.8^a^ (0.9)
Nonjudging of inner experience (*n* = 33)	25.8 (1.1)	29.0^a^ (1.1)	28.2 (1.1)
Nonreactivity to inner experience (*n* = 33)	19.9 (0.6)	23.5^a^ (0.6)	22.2^a^ (0.7)
Observing (*n* = 32)	25.5 (0.8)	30.0^a^ (0.9)	28.9^a^ (0.9)
Describing (*n* = 34)	27.7 (1.0)	29.7^a^ (0.9)	29.6^a^ (0.9)

All significant differences survived Bonferroni corrections. Higher scores on all measures indicate a higher level of the construct being measured, such as higher distress or higher mindfulness.

^a^ Indicates values differing significantly from baseline, *p* < 0.05.

^b^ Indicates values differing significantly from post-MIHP, *p* < 0.05.

Exp, experience; MIHP, Mindfulness for Interdisciplinary Health Care Professionals; SE, standard error.

At follow-up, exhaustion was the only impairment outcome that maintained a significant improvement from baseline. In fact, cynicism and perceived stress returned to baseline levels at the follow-up time period. Four of the five mindfulness skills maintained a significant improvement from baseline to follow-up: acting with awareness, nonreactivity to inner experience, observing, and describing. At the follow-up, nonjudging of inner experience was not significantly different than baseline or post-MIHP.

The percentage of participants who exhibited reliable change varied by measure and is indicated in Table 3.

**Table 3.** Reliable Change Scores

	Baseline to post-MIHP			Post-MIHP to follow-up		
Measure	Nonreliable change (%)	Reliable improve (%)	Reliable decline (%)	Nonreliable change (%)	Reliable improve (%)	Reliable decline (%)
Exhaustion	57	34	9	43	46	11
Cynicism	66	29	6	71	14	14
Perceived stress	41	56	3	63	25	13
Acting with awareness	59	38	3	69	31	0
Nonjudging of inner experience	55	39	6	53	35	12
Nonreactivity to inner experience	74	27	0	76	18	6
Observing	47	50	3	73	27	0
Describing	74	27	0	77	21	3

Percentages for RCI are calculated from the valid total *n* for each measure. For distress and impairment outcomes, improvements are interpreted as a decrease. For mindfulness skills, improvements are interpreted as an increase.

Change score correlations between impairment outcomes and mindfulness skills are presented in Tables 4 and 5. From baseline to post-MIHP, change in acting with awareness and nonjudging of inner experience were negatively associated with change in exhaustion and perceived stress over the same time period. Changes in nonreactivity to inner experience and describing were not correlated with change in any distress or impairment outcome. From baseline to post-MIHP, change in observing was positively associated with change in cynicism at the same time points.

**Table 4.** Change Score Correlations from Baseline to Post-Mindfulness for Interdisciplinary Health Care Professionals

	Act aware	Nonjudge	Nonreact	Observe	Describe
Exhaustion	−0.61^**^	−0.41^*^	−0.29	0.01	0.07
Cynicism	−0.35	−0.34	0.13	0.41^*^	−0.07
Perceived stress	−0.47^*^	−0.47^**^	−0.11	−0.01	−0.13
Activity impairment	−0.20	−0.11	0.03	0.16	0.13

^*^ Indicates correlation is significant at the 0.05 level (two tailed).

^**^ Indicates correlation is significant at the 0.01 level (two tailed).

Act aware = acting with awareness; nonjudge = nonjudging of inner experience; nonreact = nonreactivity to inner experience; observe = observing; describe = describing.

**Table 5.** Change Score Correlations from Post-Mindfulness for Interdisciplinary Health Care Professionals to Follow-Up

	Act aware	Nonjudge	Nonreact	Observe	Describe
Exhaustion	−0.65^**^	−0.08	−0.13	0.06	−0.20
Cynicism	0.15	−0.51^**^	−0.17	−0.11	−0.43^*^
Perceived stress	−0.57^**^	−0.27	−0.19	−0.32	−0.21
Activity impairment	−0.41^*^	−0.40^*^	−0.59^**^	−0.02	−0.05

^*^ Indicates correlation is significant at the 0.05 level (two tailed).

^**^ Indicates correlation is significant at the 0.01 level (two tailed).

Act aware = acting with awareness; nonjudge = nonjudging of inner experience; nonreact = nonreactivity to inner experience; observe = observing; describe = describing.

For the second time period, post-MIHP to follow-up, change in acting with awareness was negatively associated with change in exhaustion, perceived stress, and activity impairment. Change in nonjudging of inner experience was negatively associated with change in cynicism and activity impairment. Change in nonreactivity to inner experience was negatively correlated with change in activity impairment, and change in describing was negatively associated with change in cynicism. Change in observing was not significantly correlated with change in any distress or impairment outcome.

## Discussion

This study sought to address gaps in the literature by investigating the maintained effects of mindfulness intervention for HCP students, MIHP, over a 3-month follow-up time period and used RCIs to explore individual-level response. Then, correlations among changes in mindfulness skills and work-relevant outcomes were investigated to determine possible mechanisms by which MIHP may have its effects.

The investigation of outcomes at the follow-up revealed burnout and four key mindfulness skills maintained improvement over time, suggesting possible sustained effects in these outcomes. Specifically, one subscale of burnout (exhaustion) maintained improvement, and four of the five mindfulness skills demonstrated maintained improvement from post-MIHP to follow-up. Nonjudgment of inner experiences was the only mindfulness skill that did not show sustained effects. Although these findings are encouraging, when compared with previous work evaluating MIHP at follow-up,^24^ these results demonstrate less maintenance of effects on distress outcomes and impairment.

This could be due to a larger sample than that observed in previous work, thus more variance in outcomes. These results suggest a need for booster sessions, along with institutional support to incorporate MIHP into curriculum, to improve upon long-term effects in distress and impairment. In line with previous work,^24^ nearly all the mindfulness skills demonstrated maintained increases at the follow-up, suggesting that despite a trend for distress and impairment outcomes to return to baseline levels, participants retained gains in mindfulness skills. MIHP may train these skills, producing long-lasting effects, but in the absence of booster sessions and institutional support for continued mindfulness practice, these skills may not translate into protection against HCP-relevant stress outcomes.

Reliable change analyses found that overall outcomes demonstrated very little deterioration over time, contributing to the evidence that MIHP has potentially protective effects in this population of HCP students. Nevertheless, there was clearly a large subset of participants who did not reliably improve on the outcomes of interest following MIHP. It is likely that many factors interfered with improvements in perceived distress, impairment, and mindfulness skills, including historical artifacts, timing in the students' schedules (e.g., end of semester and concurrence with final examinations), poor adherence, and personal stressors.

In addition, the calculation of reliable change is limited by the quality of published norms for measures used, which may not fully reflect the current sample. Future research could make use of qualitative methods to better understand the subtleties of responder status to optimize the intervention for HCP students. Within that context, a quarter or more of participants demonstrated reliable improvement in all the outcomes from pre- to post-MIHP and nearly all outcomes at the follow-up.

More research could investigate the characteristics of those that demonstrated little reliable change as well as of those that demonstrated reliable improvements across outcomes to better characterize those for whom MIHP may be particularly beneficial, as well as identify treatment targets or ideal characteristics for future study procedures.

We also aimed to investigate the relationships between changes in mindfulness skills and work-relevant outcomes. Acting with awareness and nonjudging of inner experience were consistently related to distress and impairment outcomes across time periods. These specific skills may be constructs that act as active ingredients in the process by which MIHP has its effects on work-relevant outcomes. Awareness with a nonjudgmental attitude is a repeated theme in MIHP and may be a vital aspect of this intervention. Qualitative methods could further explore these findings by exploring participants' perception of the intervention's active ingredients.

Investigation of associations between mindfulness skills with distress and impairment outcomes found a greater number of associations at the 3-month follow-up, as compared with the number of these associations during the intervention period. This pattern of results may suggest that as intervention effects decay, the maintenance of mindfulness skills may be particularly important for protecting against HCP-related distress and impairment. These results echo findings in non-HCP samples. One study found that benefits following a mindfulness-based intervention were mediated by dispositional mindfulness, as well as self-compassion and worry.^48^ A meta-analysis found that mindfulness skills were significant mediators of mental health outcomes in non-HCP samples.^49^ Within the context of the previous literature, our results suggest that changes in mindfulness skills may be mechanisms by which work-relevant outcomes improve following MIHP.

An unexpected finding revealed a positive correlation between change in the skill of observing and change in the burnout subscale cynicism, signifying that improvement in this mindfulness skill coincided with increased cynicism. This finding may be explained by previous work evaluating the five facets of mindfulness. Specifically, studies have found that the observing facet is often characteristically different than the other skills. In nonmeditating samples, observing is often positively correlated with poorer outcomes; however, this pattern is not present in experienced meditators.^19,50–53^ It may be that in this sample of HCP students, observing can occur *without* other key mindful qualities, such that observations may have a maladaptive effect in the absence of open awareness or nonjudgment.^53^

This study is not without limitations. Without a control group on which to compare these analyses, the interpretations are limited and cannot infer causality. The use of correlations between change scores also limits our ability to make conclusions regarding mechanism of effects. All the outcomes investigated in this study were self-reported, and thus do not capture objective changes in mindfulness, attention, or stress. More ecologically valid assessments of distress, impairment, and mindfulness, as well as multimodal assessments of these constructs, would advance our understanding of responder status and mechanisms by which MIHP has its effects.

## Conclusion

Taken together, the mindfulness skills acquired following MIHP appear to be important intervention targets that demonstrate reliable improvements over time, and may be mechanisms by which improvements in distress and impairment occur. Our findings supplement previous work with evidence that acquired mindfulness skills may be vital to the ongoing protective effects of MIHP on distress and impairment outcomes. Future research should employ qualitative methodology to investigate how and for whom MIHP is most helpful, why it may not be beneficial for some, and how to optimize its active ingredients.

## Data, Materials, and/or Code Availability

The data that support the findings of this study are available from the corresponding author upon reasonable request.

## Abbreviations Used

HCP: health care professional
MIHP: Mindfulness for Interdisciplinary Health Care Professionals
RCI: reliable change index
RCT: randomized controlled trial
